# Self-Concept Clarity and Learning Engagement: The Sequence-Mediating Role of the Sense of Life Meaning and Future Orientation

**DOI:** 10.3390/ijerph20064808

**Published:** 2023-03-09

**Authors:** Yafei Liu, Siyu Di, Yixianzhi Zhang, Chao Ma

**Affiliations:** 1 Normal College, Shihezi University, Shihezi 832000, China; 2 Center of Application of Psychological Research, Shihezi University, Shihezi 832003, China

**Keywords:** self-concept clarity, learning engagement, sense of life meaning, future orientation

## Abstract

In this study, we systematically examined the effects of self-concept clarity on high school students’ learning engagement and the mediating role of sense of life meaning and future orientation between self-concept clarity and learning engagement in order to provide guidance to enhance students’ learning engagement. A total of 997 students from freshmen to seniors were selected for the study using a cluster random sampling method. The following tools were used: the Self-Concept Clarity Scale, the Learning Engagement Scale, the Sense of Life Meaning Scale, and the Future Orientation Questionnaire. The results indicated that the clarity of self-concept positively predicted the level of learning engagement of high school students. Sense of life meaning and future orientation partially mediated the effect between self-concept clarity and learning engagement, and sense of life meaning and future orientation had sequence-mediated effects between self-concept clarity and learning engagement among high school students. This study suggests that high levels of self-concept clarity can help high school students actively seek a sense of life meaning and make more optimistic future orientations, thereby increasing their level of learning engagement.

## 1. Introduction

Improving the quality of talent cultivation is an inevitable choice to promote the high-quality development of education [1]. However, the highly globalized and digitized society of the future is driving the emphasis on talent development from the cultivation of students’ cognitive factors to a focus on students’ access to learning and the learning process [2,3]. Learning engagement, as an important variable that directly affects student learning acquisition, is becoming an increasingly important issue in research in the international education field [4]. Along with the “pan-entertainment” of online information, spiritual life is gradually alienated to the individual’s pursuit of entertainment life. At the same time, learners’ weak self-control and lack of emotionality have led to a low level of engagement in learning and an increasing dropout rate year by year [5,6]. Therefore, it is necessary to study the influencing factors of learning engagement in depth so as to provide guidance to enhance students’ learning engagement.

Learning engagement refers to uninterrupted motivation and an optimistic emotional state that an individual exhibits during the learning process [7]. As a key indicator of the quality of student learning, learning engagement not only enhances students’ learning persistence and confidence but is also an important variable in predicting academic performance and achievement [8]. High school students are in the middle of adolescent development, an important stage of physical and mental growth, as well as knowledge seeking and learning [9]. Studies have found that adolescents are prone to exhibit lower levels of learning engagement in the middle stages of development compared to the early stages, which are predominantly driven by the external environment, and the later stages, which are primarily driven by the internal self [10]. The reason for this is that individuals in this period are faced with both an ambiguous ego and complex environments; therefore, how to obtain a balance between the two is an important issue affecting learning engagement. However, existing studies focus on examining the complex influence of the external environment on students’ learning engagement, such as family socioeconomic status [11], parenting style and educational expectations [12], the classroom environment [13], and teacher–student interaction in online learning [14]. At the individual level, the focus is mainly on how to motivate students to learn in terms of learning efficacy [15]. However, the construction of individual self-integrity is neglected. Therefore, it is necessary to reveal the influence process of learning engagement from the perspective of constructing the inner identity of the high school student’s self so as to promote the unity of the individual’s mind and body and to motivate their learning in the most effective manner possible [16].

As a key part of the self -concept structure and a major component of self-awareness [17], self-concept clarity refers to the clarity of an individual’s beliefs about themselves and emphasizes the internal consistency and stability of self-understanding [18]. It has a positive effect on optimizing student learning engagement [10]. The “expectancy-value” theory of motivation suggests that the individual’s self-concept and self-relevant beliefs play an integral role in motivational development and are decisive factors in the choice of achievement behavior and subsequent performance [19]. Research has demonstrated that students’ engagement in learning activities, including cognitive and affective engagement, is driven by students’ motivational characteristics such as self-concept [20]. Students with high self-concept clarity report more engagement in learning than students with low self-concept clarity [21,22,23]. As a corollary, self-concept clarity has an important impact on learning engagement, and it is important to examine the mechanisms underlying the relationship between self-concept clarity and learning engagement.

The concept of sense of life meaning is the perception and experience of the purpose, value, and significance of life [24]. Explicit self-concept plays a central role in the experience of the sense of life meaning for adolescence and adulthood [24]. The identity capital model suggests that individuals with high self-concept clarity gradually clarify the goals of their life and develop the means to achieve them as they continually reflect on their life experiences [25]. Research has demonstrated that individuals with higher levels of self-concept clarity place greater value on giving their lives a sense of meaning [26]. Conversely, individuals with lower levels of self-concept clarity tend to neglect the value of assigning a sense of meaning to their lives and even develop negative perceptions about why life exists. In addition, purpose is an important part of the sense of life meaning, and a strong sense of purpose can lead people to focus on what is most important to them [27]. Research has found that actively seeking a sense of life meaning can be effective in evoking motivation in students [28]. Furthermore, learning motivation, as a key factor influencing learning engagement, can directly predict the degree of learning engagement [29].

Future orientation refers to an individual’s cognitive preferences for future action, subjective affective experience, and tendency to act on future volition [30]. The idea of “value-control” theory is that future orientation encourages people to actively analyze the benefits of environmental resources in order to plan for the future rather than passively accepting environmental influences, therefore exhibiting proactive learning behaviors [31]. Empirical research shows that individuals with a positive future orientation maintain a strong intrinsic motivation to achieve their goals and that they do not accept the influence of the environment in a passive way but that learning is valuable and requires more of their time and effort based on an active analysis of external environmental resources and their own characteristics [31,32]. Individuals with a positive future orientation also tend to plan and manage their time more rationally and effectively and prefer to choose to delay rewards to ensure that more productive learning can be maintained [33,34]. In addition, Davis’ “cognitive-behavioral” model suggests that self-concept clarity, as a cognitive attribute of the self, provides a future-oriented way of thinking that regulates and motivates individuals’ attitudes and behaviors [35]. For example, a cluster analysis study showed that individuals with stable and consistent self-concept clarity maintain a more positive future orientation and are more likely to actively plan for the achievement of long-term future goals [36]. However, individuals with non-stable self-concept clarity hold a more pronounced negative view of the future [37].

As mentioned above, clarity of self-concept may influence high school students’ learning engagement through a sense of life meaning and future orientation. Does self-concept clarity also play a role in future orientation through sense of life meaning, which, in turn, affects the learning engagement of high school students? The “expansion-construction of positive emotions” theory suggests that positive emotions induce and extract more meaning-related feedback while expanding the scope of an individual’s thinking activities and increasing cognitive flexibility, leading the individual to store more resources in a short period of time [38] These resources act as a positive buffer when individuals are planning for the future, helping them to maintain a stable and healthy mental state and to plan and prepare for the future [38]. As an indispensable positive psychological resource, sense of life meaning can effectively awaken positive emotions in individuals. Related research shows that people with a positive sense of life meaning have a greater sense of happiness and can have more positive emotional experiences [39]. This positive emotion makes them have more positive perceptions and emotions about the future so that they are also willing to make more willful efforts for the future. From a positive psychology perspective, researchers have also found that experiencing a positive sense of life meaning can effectively enhance an individual’s ability to cope with stress in the future [40,41]. For example, Miao and Gan explored the potential mechanism of the effect of the experience of a sense of life meaning on future orientation options through a longitudinal study and found that experience of a sense of life meaning can drive individuals to engage in resource accumulation and promote their ability to cope with future stressful events [42].

**Figure 1:**
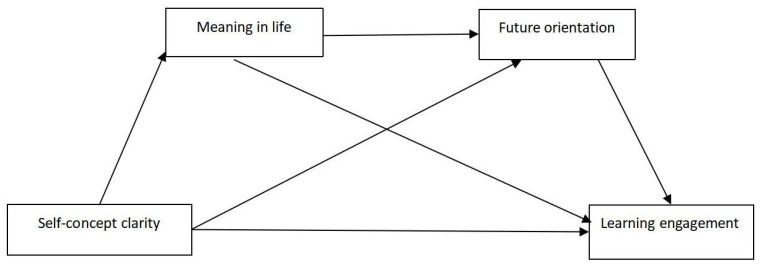
The conceptual multiple mediation model.

In summary, the present study proposes a sequence mediation model by incorporating sense of life meaning and future orientation variables. Through this study, we attempt to reveal the sequential characteristics that the two mediating variables, sense of life meaning and future orientation, exhibit in the relationship between self-concept clarity and learning engagement. Sequential mediation reveals more complex mechanisms of the relationship between predictor and outcome variables than traditional simple or multiple mediators; therefore, examining the sequential mediating role of both sense of life meaning and future orientation can provide insight into the mechanisms underlying the prediction of learning engagement via self-concept clarity. The proposed model is illustrated in Figure 1.

## 2. Materials and Methods

### 2.1. Participants

A total of 1084 high school students from five general senior high schools in two cities in western China were selected for the survey by whole-group convenience sampling. Researchers and school classroom teachers cooperated in issuing questionnaires to the participants in classroom settings. All participants provided their informed consent and spent approximately 15 min completing each questionnaire item. After excluding invalid questionnaires, a total of 997 questionnaires were collected (an effective response rate of 91.97%). Of the responses, 435 (43.6%) were from boys, and 562 (56.4%) were from girls. Among the 997 high school students, 345 (34.6%) were from the senior class, 351 (35.2%) were from the sophomore class, and 301 (30.2%) were from the junior class. Of the 997 participants, 437 (43.8%) were only children, and 558 (56.2%) were not only children. In terms of the education level of fathers and mothers, 18.8% and 28.6% had completed elementary school or less, 42.9% and 35.7% had completed junior high school, 26.5% and 21.6% had completed high school or junior college, 7.0% and 9.3% had a college or bachelor’s degree, and 4.8% and 4.8% had a master’s degree or above, respectively. Among respondents, there were 603 (60.5%) urban residents and 394 (39.5%) rural residents. The mean age of the subjects was 16.33 ± 1.01 years. The study was conducted in accordance with the Declaration of Helsinki and was approved by the Science and Technology Ethics Committee of the First Affiliated Hospital of Shihezi University School of Medicine.

### 2.2. Materials

#### 2.2.1. Self-Concept Clarity

The short version of the Self-Concept Clarity Scale was used to assess the clarity of adolescents’ self-concept [43]. The scale consists of 12 items. The participants answered the items on a seven-point Likert scale (1 = very inconsistent, 7 = very consistent). The higher the score on the scale, the higher the degree of clarity of self-perception. The scale has positive reliability and validity. In the present study, the Cronbach’s α was 0.74.

#### 2.2.2. Sense of Life Meaning

The level of sense of life meaning was assessed using the Sense of Life Meaning Scale [44]. The scale consists of 10 items that include two dimensions, which are the sense of having meaning and the sense of seeking meaning. Among them, sense of having meaning focuses on the extent to which individuals understand and perceive the meaning of life, while sense of seeking meaning focuses on the individual’s drive to actively pursue meaning. Participants answered each item on a seven-point Likert scale (1 = very unlikely to meet, 7 = very likely to meet). Higher scale scores indicate higher levels of meaning in an individual’s life. The scale has positive reliability and validity. In this study, Cronbach’s α was 0.85, while those of the two dimensions constituting the scale were 0.80 and 0.87, respectively.

#### 2.2.3. Future Orientation

The Future Orientation Questionnaire for Adolescents was used to measure future orientation [45]. The questionnaire consists of 31 items and includes three dimensions: future cognition, future affect, and future volitional action. The participants answered the items using a five-point Likert scale (1 = not at all, 5 = complete). The higher the scale score, the higher the level of development of the individual’s future orientation. The scale has positive reliability and validity. In this study, Cronbach’s α was 0.89, while those of the three dimensions constituting the scale were 0.82, 0.86, and 0.78, respectively.

#### 2.2.4. Learning Engagement

Learning engagement was measured by the Learning Engagement Scale [46]. The scale includes three dimensions: dedication, vigor, and concentration. Dedication refers to an individual’s willingness to commit to learning as a trailblazer. Vigor refers to an individual’s willingness to devote energy to learning and to persevere despite difficulties. Dedication implies that the individual is fully focused on learning and is willing to devote a significant amount of time to it [47]. The scale consists of 17 items. Participants answered the items on a seven-point scale (1 = never, 7 = always). Higher scale scores indicate higher levels of individual commitment to learning. The scale has positive reliability and validity. In this study, Cronbach’s α was 0.95, while those of three dimensions constituting the scale were 0.90, 0.90, and 0.91, respectively.

### 2.3. Data Analysis

Data processing was first conducted using Epidata 3.1. (The EpiData Association, Odense, Denmark). Next, IBM SPSS Statistics version 21.0 (IBM SPSS Inc., Chicago, IL, USA) was used to perform reliability analysis, descriptive analysis, common method deviation analysis, and correlation analysis for each predictor variable. Finally, Model 6 in the PROCESS v.3.5 (Andrew F. Hayes, Calgary, AB, Canada) macro program and the bias-corrected percentile bootstrap method were used for mediating effects analysis.

## 3. Results

### 3.1. Common Method Bias Test

A common method deviation test was conducted using Harman’s one-way method [48]. Each entry of self-concept clarity, sense of life meaning, future orientation, and learning engagement was included in an exploratory factor analysis. The results showed that there were 13 common factors with characteristic roots greater than one; the first factor explained 23.60% of the total variance, which was not higher than the critical level value of 40%. Therefore, the deviation from the common method of this study was considered to be within the acceptable range.

### 3.2. Behavioral Results

Table 1 shows the mean value, standard deviation, and correlation coefficient of each variable. The results of the descriptive analysis and the correlation matrix of each predictor variable showed that the predictor variables of self-concept clarity, sense of life meaning, future orientation, and learning engagement were significantly and positively correlated with each other.

### 3.3. Mediation Analyses

In this study, testing the sequence mediation effect requires two steps, namely a simple regression analysis followed by the construction of a sequence mediation model [49]. In this study, prior to analysis, correlation analysis was first performed on demographic variables such as gender, age, grade level, and whether or not the student was an only child, as well as predictor variables, such as self-concept clarity, sense of life meaning, future orientation, and learning engagement. The analysis did not reveal a significant correlation between demographic variables and each predictor variable. Therefore, demographic variables were not included when performing simple regression analysis and constructing sequence mediation models.

In the first step, regression analysis was performed with self-concept clarity as the independent variable, learning engagement as the dependent variable, and sense of life meaning and future orientation as the mediating variables. The results showed that self-concept clarity significantly and positively predicted high school students’ learning engagement (β = 0.33, *p* < 0.01). After adding the mediating variables sense of life meaning and future orientation to the regression equation, self-concept clarity not only significantly and positively predicted high school students’ sense of life meaning (β = 0.25, *p* < 0.01) but also their future orientation (β = 0.14, *p* < 0.01). The direct effect of self-concept clarity on learning engagement was also significant (β = 0.12, *p* < 0.05). Sense of life meaning not only significantly and positively predicted high school students’ future orientation (β = 0.31, *p* < 0.01) but also significantly and positively predicted learning engagement (β = 0.43, *p* < 0.01). Future orientation significantly and positively predicted high school students’ learning engagement (β = 0.47, *p* < 0.01). The results of the analysis are shown in Table 2.

**Figure 2:**
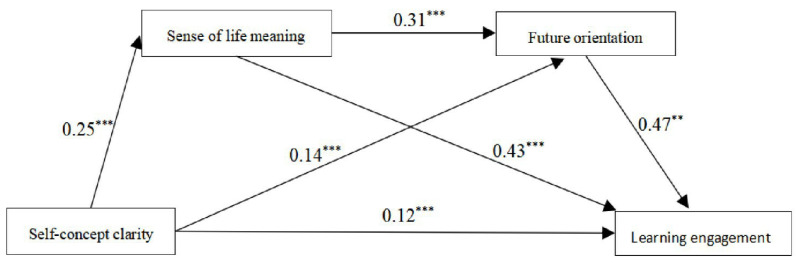
A multiple mediation model for the association between self-concept clarity and learning engagement. ** *p* < 0.01; *** *p* < 0.001.

In the second step, the significance of the mediating effect of sense of meaningfulness of life and future orientation was tested using the bias-corrected percentile bootstrap method. The study was set up with 5000 replicate samples, and the sign of the upper and lower 95% confidence interval was observed; if the signs were the same, the mediating effect was statistically significant. The results show that the model has a significant sequence-mediating effect, with a total indirect effect value of 0.21, accounting for 63.64% of the total effect. The mediating effect consisted of three parts. Indirect effect 1 (Effect = 0.11) consisted of the path “self-concept clarity → sense of life meaning → learning engagement”, which accounted for 33.33% of the total effect. Indirect effect 2 (Effect = 0.03) consisted of the path “self-concept clarity → future orientation → learning engagement”, accounting for 21.21% of the total effect. Indirect effect 3 (Effect = 0.04) consisted of the path “self-concept clarity → sense of life meaning → future orientation → learning engagement”, accounting for 9.10% of the total effect. In addition, the upper and lower limits of the 95% confidence interval for all three indirect effects were positive, indicating that all indirect effects were statistically significant. Furthermore, to test the variability between paths, a two-by-two comparison of the indirect effect paths was performed using model 6. Comparisons 1 and 3 show that indirect effect 1 and indirect effect 2, as well as indirect effect 2 and indirect effect 3, both contain 0 in the 95% confidence interval, indicating that there is no significant difference between these two groups. In contrast, comparison 2 shows that indirect effect 1 and indirect effect 3 do not contain 0 in the 95% confidence interval, which implies a significant difference between indirect effect 1 and indirect effect 3. The specific results of the analysis are shown in Table 3 and Figure 2.

## 4. Discussion

To explore the intrinsic influence mechanism of learning engagement of high school students from the perspective of constructing individual self-identity, in this study, we developed a sequence mediation model with the aim of examining whether there is a sequence mediation effect of sense of life meaning and future orientation between self-concept clarity and learning engagement. This not only helps to reveal the influence of self-concept clarity on individual learning engagement levels and its intrinsic mechanisms but can also provide empirical evidence and suggestions for cultivating and shaping individual self-identity.

The current findings suggests that self-concept clarity is positively related to learning engagement. Based on previous research on the influence of self-concept on learning engagement, the results of this study further suggest that self-concept clarity positively influences high school students’ learning engagement, which extends previous research findings. According to the “expectancy-value” motivation theory, self-concept clarity, as one of the important elements of the self-concept system, provides a motivational basis for promoting high school students’ learning engagement and positively influences their learning behaviors [50]. It has been found that lower self-concept clarity results in individuals exhibiting higher levels of self-concept conflict or confusion [10]. This state of confusion or conflict threatens self-consistency and self-integrity and even leads to self-contradiction, which, in turn, depletes self-control resources and affects ensuing learning engagement behaviors [51]. Conversely, adolescents with more stable self-concepts present a more consistent self [52].

High school students are at a critical time in their self-awareness development. At this time, students with a consistent, stable, and clear self-concept are more likely to be motivated to learn, maintain a high level of goal persistence, and focus more attention on their current learning behaviors. Therefore, the results of this study reveal the importance of constructing high school students’ self-identity to promote their learning engagement.

We also found that sense of life meaning mediates the relationship between self-concept clarity and learning engagement. This study revealed that clarity of self-concept affects high school students’ learning engagement indirectly through sense of life meaning. Sense of life meaning is an individual’s view of the purpose and value of his or her existence [53]. On the one hand, a sense of life meaning stems from an individual’s understanding of the self, the environment, and the relationship between the two. High school students with higher self-concept clarity are better at integrating their daily experiences, and can develop a coherent and consistent understanding of life based on the values they have established about life events and experiences, which, in turn, enhances their perception of the meaning of life [54]. On the other hand, Beumeister argues that people consider themselves to be “living meaningfully” when all four of the following needs are met: acquiring meaning, clarifying their values, developing a sense of efficacy, and having a sense of self-worth, which are potential drivers for individuals to seek meaning in their own lives [55]. This is a potential driver for individuals to seek meaning in their lives. High levels of self-concept clarity indicate that individuals have more self-awareness and a richer sense of fulfillment of these four needs, resulting in a strong inner being and a constant source of motivation to encourage high school students to invest more consistently in meaningful pursuits [56,57].

The results indicate that future orientation is another important mediator between self-concept clarity and learning engagement. This study revealed that self-concept clarity affects high school students’ learning engagement indirectly through future orientation. Adolescents with stable and consistent self-concept clarity are more optimistic and have a longer-term view of the future [58]. High school students are in a critical period of self-awareness development, and stable and consistent self-awareness motivates them to be clear about their future goals and to be actively prepared to achieve them. Positive future orientation can also influence high school students’ academic psychology and behavior [58]. Students with a positive future orientation are more willing to invest more effort in learning activities and are relatively more persistent [59].

This study revealed that sense of life meaning and future orientation play a sequence-mediating role between self-concept clarity and learning engagement among high school students. This suggests that self-concept clarity facilitates high school students’ perceptions of the meaning of life and motivates them to make more future orientations and then actively engage in learning behaviors. Specifically, greater clarity of self-concept implies that individuals have a relatively complete and comprehensive perception and evaluation of themselves, which influences their perception and experience of their own existence in life. That is, individuals who view themselves in a positive light will also view the value of their life existence in a positive light, therefore better perceiving and experiencing the meaning of life and getting rid of the anxiety caused by emptiness and helplessness. At the same time, the more positive high school students feel about the meaning of their lives, the more emotionally charged they will be in the future and the more powerful they will be in their future actions, thereby reducing academic burnout, maintaining more adequate academic motivation, and engaging in the entire learning process in a state closer to “immersion”, ultimately achieving good academic results. In contrast, a blurred self-concept means that high school students cannot form stable and consistent self-beliefs, cannot perceive the meaning and value of their own existence, and have lower hopes and expectations for the future, which makes it difficult for them to plan their time effectively and reasonably and choose to delay gratification, resulting in a lack of commitment to learning and lower academic achievement throughout the learning process. The results of this study further reveal the importance of strengthening the education of life and future orientation of high school students while building their self-identity to enhance learning engagement and improve learning quality.

In addition, by examining the differences between the indirect pathways, in this study, we found that there was a significant difference between the separate mediating effect of sense of life meaning and the sequential mediating effect of sense of life meaning and future orientation. Furthermore, the separate mediated effect of sense of life meaning was greater than the sequential mediating effect of sense of life meaning and future orientation. This further indicates the important role of sense of life meaning in self-concept clarity affecting high school students’ learning engagement. Previous research revealed that adolescence is an important period in the formation of self-identity during which adolescents continuously seek self-validation and explore the meaning of their lives. Meaning, on the other hand, helps people integrate their diverse and complex daily experiences and form a sense of control and coherence over the external world [60]. At the same time, students who actively seek a sense of life meaning are able to connect the search for life meaning with the understanding of the value of learning in the process of their studies. Conversely, the lack of a sense of life meaning leads students to be confused with respect to a deep understanding of life meaning and learning value, resulting in a lack of enthusiasm and concentration in their studies, which, in turn, affects their learning effectiveness. This suggests that high school students should be actively guided to comprehend and construct a sense of life meaning in the study process in order to better enhance learning engagement and to form a positive interaction mechanism between self-concept clarity, sense of life meaning, and learning engagement.

## 5. Limitations and Implications

The current study has some limitations. First, the present study reveals the mechanism of the effect of self-concept clarity on high school students’ learning engagement through a cross-sectional design. Future longitudinal studies or behavioral experiments can be conducted to further verify the relationship between the relevant variables. Second, the abovementioned relationship studies are mostly based on average-level sample data, which cannot adequately reflect the heterogeneity of individual psychological and behavioral development hidden under the simple linear relationship or how it affects high school students’ learning engagement.

This study systematically and deeply reveals the psychological mechanisms by which self-concept clarity influences learning engagement through a sense of life meaning and future orientation and enriches the research on self-identity to improve the quality of learning of high school students. As high school students are in an important period of constructing self-identity and academic development, the results of this study can provide inspiration for parents and educators to improve the quality of learning of high school students. First, parents and educators should correctly understand the importance of cultivating high school students’ self-concept, guiding and encouraging high school students to actively engage in self-exploration to alleviate internal conflicts and contradictions, thereby reducing self-loss and improving their focus on learning. Secondly, schools should also incorporate the construction of life meaning into classroom teaching objectives, systematically carry out corresponding curriculum teaching, and explore positive and effective cultivation models to motivate high school students and improve their learning engagement status.

## 6. Conclusions

The findings of this study are as follows. First, self-concept clarity, sense of life meaning, future orientation, and learning engagement are two-way-correlated, and self-concept clarity is a significant positive predictor of high school students’ learning engagement. Secondly, sense of life meaning and future orientation mediate the relationship between self-concept clarity and learning engagement of high school students. The mediating effect consists of three pathways: first, a separate mediating effect of sense of life meaning; second, a separate mediating effect of future orientation; and third, a sequence-mediating effect of sense of life meaning and future orientation.

## Figures and Tables

**Table 1:** Descriptive statistics and correlation among variables (*n* = 997).

	M	SD	1	2	3	4
Self-concept clarity	2.86	0.57	1			
Sense of life meaning	4.94	1.03	0.14 **	1		
Future orientation	3.42	0.55	0.23 **	0.60 **	1	
Learning engagement	4.19	1.13	0.17 **	0.54 **	0.48 **	1

Note: ** *p* < 0.01; “M” denotes the mean. “SD” denotes standard deviation.

**Table 2:** The pathways of the multiple mediation model.

Regression Equation		Overall Fitting Index			Significance of the Regression Coefficient		
Outcome Variable	Predictive Variable	R	R^2^	F	β	95%CI	t
Learning engagement	Self-concept clarity	0.17	0.03	28.34 ***	0.33	[0.21, 0.45]	5.32 ***
Sense of life meaning	Self-concept clarity	0.14	0.02	19.55 ***	0.25	[0.14, 0.36]	4.42 ***
Future orientation	Self-concept clarity	0.62	0.39	313.72 ***	0.14	[0.10, 0.19]	5.96 ***
	Sense of life meaning				0.31	[0.28, 0.34]	23.27 ***
Learning engagement	Self-concept clarity	0.57	0.33	161.79 ***	0.12	[0.01, 0.22]	2.23 *
	Sense of life meaning				0.43	[0.36, 0.50]	11.92 ***
	Future orientation				0.47	[0.34, 0.61]	6.91 ***

Note: * *p* < 0.05; *** *p* < 0.001.

**Table 3:** Analysis of sequence-mediating effects of sense of life meaning and future orientation.

	Effect	Boot SE	95%CI	Relative Mediation Effect
Direct effect: self-concept clarity–learning engagement	0.12	0.05	[0.01, 0.22]	
Total indirect effect: Ind1 + Ind2 + Ind3	0.21	0.04	[0.13, 0.30]	63.64%
Ind1: Self-concept clarity–sense of life meaning–learning engagement	0.11	0.03	[0.05, 0.17]	33.33%
Ind2: Self-concept clarity–future orientation–learning engagement	0.07	0.02	[0.04, 0.11]	21.21%
Ind3: Self-concept clarity–sense of life meaning–future orientation–learning engagement	0.03	0.01	[0.02, 0.06]	9.10%
Comparison 1: Ind1–Ind2	0.04	0.04	[−0.04, 0.11]	
Comparison 2: Ind1–Ind3	0.07	0.03	[0.03, 0.12]	
Comparison 3: Ind2–Ind3	0.03	0.02	[0.00, 0.07]	

Note: Effect and BootSE represent the effect values and standard errors of indirect effects estimated by the percentile bootstrap method, respectively.

## Data Availability

The data presented in this study are available upon request from the corresponding author. The data are not publicly available due to concerns about privacy and ethics in personal decision making.
